# Shifting Beliefs about Suicide: Pre-Post Evaluation of the Effectiveness of a Program for Workers in the Construction Industry

**DOI:** 10.3390/ijerph15102106

**Published:** 2018-09-25

**Authors:** Tania L. King, Jorgen Gullestrup, Philip J. Batterham, Brian Kelly, Chris Lockwood, Helen Lingard, Samuel B. Harvey, Anthony D. LaMontagne, Allison Milner

**Affiliations:** 1 Centre for Health Equity, School of Population and Global Health, University of Melbourne, Melbourne 3010, Australia; allison.milner@unimelb.edu.au; 2 MATES in Construction, Spring Hill 4000, Australia; jorgen@micqld.org.au (J.G.); clockwood@micaus.org.au (C.L.); 3 Centre for Mental Health Research, Research School of Population Health, Australian National University, Canberra 0200, Australia; Philip.Batterham@anu.edu.au; 4 School of Medicine and Public Health, University of Newcastle, Newcastle 2308, Australia; brian.kelly@newcastle.edu.au; 5 Construction Work Health and Safety Research @ RMIT, School of Property, Construction and Project Management, RMIT University, Melbourne 3000, Australia; helen.lingard@rmit.edu.au; 6 Black Dog Institute, Faculty of Medicine, University of New South Wales, Sydney 2052, Australia; s.harvey@unsw.edu.au; 7 Work, Health and Wellbeing Unit, Population Health Research Centre, School of Health & Social Development, Deakin University, Geelong 3217, Australia; tony.lamontagne@deakin.edu.au

**Keywords:** mental health, suicide, occupation, construction workers, beliefs, intervention

## Abstract

Suicide is a significant health problem that is known to disproportionately affect those employed in manual occupations, including construction workers and tradespeople. Universal General Awareness Training (GAT) was part of a multi-component suicide prevention program in the Australian construction industry. The program’s aims were to increase awareness of mental health and suicide, reduce stigma, and encourage help-seeking and help-offering behaviours. This paper sought to examine the effectiveness of the GAT program in shifting suicide beliefs. Pre- and post-training survey data of 20,125 respondents was obtained from a database of GAT evaluation results between 2016 and 2018. Generalized estimating equation (GEE) models were fitted to examine belief changes, and predictive margins and their SEs were computed. Mean differences in belief change were obtained for the overall sample, and by occupation. Modest but significant favourable shifts in three of the four beliefs assessed were observed following GAT. Managers and professionals showed greater propensity to shift beliefs, and Labourers and Machinery Operators and Drivers showed least. Results suggest that GAT can successfully shift some beliefs regarding suicide and mental health at least in the short term, but highlight the need to tailor communication to vulnerable occupational groups.

## 1. Introduction

Across the world, suicide is a significant problem that is recognised by the World Health Organisation as a public health priority [1]. Within high income countries, it is evident that certain population groups are at higher risk of suicide than others. Men are known to be at higher risk [2], as are those employed in certain occupations [3]. Evidence of occupational patterning of suicide behaviour has catalysed an increasing focus on the workplace as a point of investigation as both a source of risk for suicide, and as a point of intervention [4]. Those in the highest occupational skill level group tend to have a lower risk of suicide, while those employed in the lowest skill level such as labourers, cleaners, or plant operators have a greater risk of suicide [3]. Within Australia, research has highlighted that those employed in manual occupations, including construction workers and tradespeople, are at higher risk of suicide than the rest of the working population [5].

Suicide is a highly dynamic and complex phenomena [6], with many influences and determinants [7,8]. Several key factors however, have been identified as precipitants of risk in the construction industry, including: job insecurity and transient working conditions, gender norms, a workplace culture that inhibits help-seeking behaviour, and stigma related to mental health conditions [9].

Perceptions and beliefs about suicide are known to inhibit help-seeking [10]. Self-stigma (self-blame, shame) is known to reduce help-seeking among those with mental illness [11,12]. While not immutable, it is known that self-stigma is difficult to shift [12,13]. There is also evidence that beliefs about treatment effectiveness or non-effectiveness serves to stymie help-seeking, as does a lack of perceived need [14]. Identifying the best means to reduce self-stigma, shift suicide beliefs and attitudes, and improve help-seeking and help-offering is critical to suicide prevention.

Suicide prevention is recognised as a major priority, and many countries have invested heavily in suicide prevention programs [15]. One method of suicide prevention that has shown some success is that of gatekeeper training [16,17]. Gatekeeper training seeks to teach specific groups the skills to recognise people at risk of suicide, and refer them to treatment or assistance [16]. Implemented in a range of settings, gatekeeper training has been identified as offering promise as a suicide prevention program, however a need for further evidence has been noted [15].

The workplace has been identified as a feasible setting in which to implement gatekeeper programs for suicide prevention [17]. Informed by this, and recognising the elevated suicide risk of construction workers, ‘MATES in Construction’ (MATES) was founded by the Building Employees Redundancy Trust (BERT) in 2008 as an industry based, workplace-focused program with the express intent to prevent suicide [18]. MATES has several key aims, including to: raise awareness about suicide in the workplace; reduce stigma associated with mental health and help-seeking; facilitate and support help-seeking and help-offering behaviours; and ensure the appropriateness and viability of help provided. The MATES program is multi-component and multi-level, and most components are delivered at construction sites or company offices. 

A key part of the MATES program is General Awareness Training (GAT) [19]. A form of gatekeeper training, GAT involves a one-hour training session and has been provided to all construction workers on work sites recruited to the study, across four states. The GAT program’s principal aim is to engage and activate construction workers in suicide prevention. GAT seeks to do this by: increasing awareness and understanding of mental health and suicide; reducing stigma associated with suicide; and encouraging helping behaviours, both in terms of help-seeking (for oneself), and help-offering (to co-workers in distress). GAT is complemented by other workplace training programs that are offered to workers once they have completed GAT [20].

Previous research has highlighted the fact that while numerous suicide interventions have been developed and implemented, few have been rigorously evaluated [14]. There is therefore, a patent need for suicide prevention efforts to be scrupulously tested. This paper aims to evaluate the effectiveness of GAT, specifically in terms of its impact on attitudes and beliefs. Shifting damaging attitudes and beliefs is core to promoting suicide awareness and understanding and is key to reducing stigma and driving appropriate help-seeking and help-offering behaviour. We hypothesised that GAT would lead to better understanding of suicide, as measured in terms of a set of beliefs.

## 2. Materials and Methods

### 2.1. Data Source

Data was from a MATES collected database of pre- and post-GAT evaluation results, collected between 2016 and 2018. Data was collected using paper surveys, with the baseline surveys conducted before training, and follow up conducted immediately afterwards. This data was collected by the program provider, with minimal burden on participants, for the purposes of on-going, general evaluation, rather than in the context of a purpose-designed evaluation research study.

### 2.2. Participants

Public and private sector construction sites across New South Wales, Western Australia, South Australia and Queensland were recruited to participate in MATES in Construction. All workers at these sites were invited to participate in GAT. Workers completing GAT are provided with a white MATES sticker to wear on their hard hat. For a worksite to be ‘MATES compliant’, all workers must be offered GAT, and an 80% training level must be maintained irrespective of staff turnover, and inclusive of sub-contracted workers (who are highly transient).

Due to the itinerant nature of the construction industry, some participants completed GAT more than once. It is likely that responses would be different for respondents completing multiple training sessions, so where a participant had completed multiple GAT sessions, we restricted analysis to participants’ first training session. While some respondents had completed up to six sessions, most of those in our analytic sample (83%) had completed GAT only once.

### 2.3. Ethics

This research was approved by the University of Melbourne Human Ethics Committee #1750927.

### 2.4. Evaluation Design

This evaluation involved an uncontrolled pre-post test design, with pre-post data linked within individuals.

### 2.5. GAT Intervention

GAT is a one-hour training session provided to all construction workers on sites recruited to the study. It is provided by MATES staff, including field officers and management. It is provided as a stand-alone session, or as a component of the Life Skills Toolbox, a training program for apprentices. GAT is delivered by two facilitators, to groups of between 20 to 200 workers. The sessions are interactive but are delivered in the style of a lecture.

This universal program aims to improve understanding of suicide and mental health so that workers are able to offer support to co-workers who display warning signs of possible increased suicide risk. It aims to do this by: increasing awareness of mental health and suicide in the industry and improving knowledge of warning signs; reducing stigma associated with suicide and mental health; and encouraging help-seeking and help-offering behaviours among workers.

### 2.6. Pre-Test Measures

Prior to GAT, participants completed a short questionnaire. This sought basic demographic information including gender, occupation, age, postcode of residence, postcode of training, as well as responses to a set of statements examining suicide and suicide prevention awareness and beliefs. (Refer to Supplementary Information for survey).

Suicide and Suicide Prevention Awareness and Beliefs

Participants indicated agreement with four statements that assessed their beliefs and awareness regarding suicide and suicide prevention. These items were central to the intention of GAT to shift attitudes and beliefs about suicide and reflected the focus of training. They were identified based on field trials of ten myths about suicide documented by the World Health Organisation in 2011 [1]. They included: Talking about suicide can cause suicide; People considering suicide often send out warning signs or invitations; Poor mental health is a workplace health and safety issue; and The construction industry must do something to reduce suicide rates. Responses were indicated on a 5-point Likert scale, from strongly agree (1) to strongly disagree (5).

For Talking about suicide can cause suicide, the desirable response was ‘strongly disagree’ (higher score), and the training program sought to shift this belief toward higher scores. For all other items, ‘strongly agree’ was the desirable response: it was therefore anticipated that the training program would shift these beliefs toward ‘strongly agree’ (lower scores).

### 2.7. Post-Test Measures

#### 2.7.1. Suicide and Suicide Prevention Awareness and Beliefs

Effectiveness of GAT was assessed at the completion of the one-hour training session, by repeating the same four statements about suicide and suicide prevention that are detailed above in the pre-test measures section.

#### 2.7.2. Experiences of Suicide and Helping Behaviours

Participants were asked three questions designed to assess their experience of suicide (knowledge of someone who had died), and experiences of help-seeking and help-offering behaviours in adverse circumstances. The response frame for these items was yes/no.

#### 2.7.3. Help-Seeking and Help-Offering Propensity

Two questions assessed likelihood of help-seeking (for themselves) and help-offering (to a friend or mate). Responses were provided on a 5-point Likert scale ranging from strongly agree to strongly disagree.

### 2.8. Covariates

**Figure 1:**
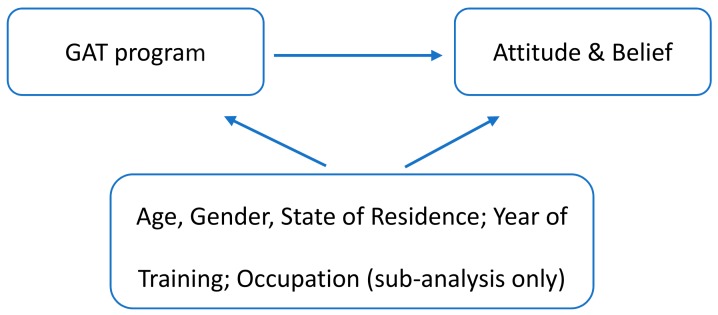
Directed acyclic graph guiding variable selection.

We adjusted for available covariates in analytic models. The selection of these was guided by a directed acyclic graph and informed by relevant literature: we chose covariates that were identified to be prior common causes of belief change, and participation in the MATES program. (See Figure 1 below). Covariates included were age (as a categorical variable, 15–24 years, 25–34 years, 35–44 years, 45 years and older), gender (male, female), and year of training (2016, 2017, 2018). MATES has been present in different states for different periods of time, so we also adjusted for state of training (New South Wales, Queensland, Western Australia, South Australia). In sub-analysis, adjustment was also made for occupational skill level (based on the Australian Standard Classification of Occupations, [21]). We used an eight category, one-digit skill level classification, however as there were no respondents in two of these categories, analysis contained six categories. We also note that apprentices, while technically not classified in the occupational grouping, were classified as Labourers.

### 2.9. Statistical Analysis

Statistical analysis was conducted using Stata, Version 15 (StataCorp LLC, College Station, TX, USA) [22].

From an eligible sample of 30,052 participants, we conducted complete case analysis and therefore excluded those with missing data on age (*n* = 2721, 9.1%), pre- and post-test beliefs and attitudes (*n* = 2689, 8.9%), and post-test experiences and expectations (*n* = 1885, 6.3%). There was no missing data on gender, state of training or training session. Data was also restricted to those completing their first GAT (*n* = 4611, 15.3% completing their second or subsequent training session were dropped from analysis). This produced a resultant sample size of *n* = 20,125. Given the considerable missing data on occupation (*n* = 12,257, 40.8% of eligible sample), this variable was excluded as a covariate in the main analysis. Sub-analysis was conducted on the smaller sample of those with complete occupation data (as well as complete data on other analytic variables, *n* = 12,853).

To examine suicide beliefs, we used the generalized estimating equation (GEE) approach [23] and fitted models using the xtgee command in Stata, with specification of an exchangeable correlation structure. GEE is an extension of generalised linear models (GLM) and enables the specification of within-group correlation structures for the panels. GEE is a commonly used method in the analysis of longitudinal data in epidemiological studies where correlated data is common [24]. GEE is also commonly used in repeated measure studies such as this, where the same subject is followed over time, and within-person observations are likely to be correlated [25]. This enabled us to account for within-person clustering, as well as within-training session clustering.

From these models, predictive margins and their SEs were computed to assess the predicted pre- and post-GAT beliefs at average covariate values. Such predictive margins are a type of direct standardization that average predicted values from regression models across the covariate distribution in the population. As mentioned, models were adjusted for age category, gender, state, year of training and clustering by training session.

To examine within person changes in beliefs by occupation, a change in belief variable was created for each belief, for each individual. This was then used to obtain mean differences in belief change, together with 95% confidence intervals for these changes.

## 3. Results

The sample was predominantly male (92.1%), and more than half of respondents were aged 25–44 years. Respondents from Queensland constituted almost half of the sample, with New South Wales being the next largest group. More than two thirds of the data collection occurred in 2017. In terms of occupational groups, Technicians and Tradeworkers were the largest group, with one quarter of respondents being classified as such. The next largest proportion of the sample were Labourers, then Managers, followed by Machinery Operators and Drivers (see Table 1).

After completing GAT, respondents were asked about their experiences of suicide and helping behaviours (both seeking and offering help), as well as their anticipated propensity to offer and seek help (see Table 2). The majority of respondents (three quarters) had known someone who had either attempted or died by suicide, and a similarly high number of respondents had assisted someone “doing it tough” (in the Australian vernacular, this means “having a hard time”). Although more than three quarters of the sample (75.1%) had helped someone else, only two fifths (39.1%) had ever sought help for themselves. When considering their propensity to seek and offer help, respondents rated themselves as extremely likely to offer help: this is reflected in the high mean (4.6 on a scale of 1–5 where 5 represents the strongest endorsement) and low standard deviation. Likelihood of anticipating seeking help for oneself in the future was notably lower, albeit still high (mean score 3.74, SD 0.92).

Table 3 presents the results of the regression analysis. In adjusted models, significant shifts in all beliefs except the first (Talking about suicide can cause suicide) were observed following GAT. The second belief targeted by GAT asked about warning signs: People considering suicide often send out warning signs or invitations, and was associated with the greatest change in belief (mean difference −0.429, 95% CI −0.45, −0.41). There was high pre-test agreement with the remaining beliefs: Poor mental health is a workplace health and safety issue (mean difference −0.155 95% CI −0.17, −0.14) and The construction industry must do something to reduce suicide rates (mean difference −0.151, 95% CI −0.16, −0.14). A significant increase in agreement was observed for both of these beliefs following training.

Sub analysis by occupation was conducted (see Table 4) on a sample of 12,853 respondents. For all occupational groups, there was little observable shift in the first belief *Talking about suicide can cause suicide*. For all other beliefs, there was a significant and positive change in beliefs for all occupational groups. Managers and Professionals and, to a slightly lesser extent, Clerical and Administrative workers were more likely to endorse these three beliefs, and also showed greater shifts in beliefs. *People considering suicide often send out warning signs or invitations* was the belief that produced greatest change, particularly among Managers, Professionals, and Clerical workers. Labourers and Machinery Operators and Drivers showed lower levels of endorsement of this belief and showed less change. This pattern was repeated for other beliefs.

Sensitivity analysis conducted using the eligible sample (rather than the analytic sample) for both the main analysis and the occupational analysis produced results consistent with those attained using the analytic sample.

## 4. Discussion

This study has shown that the gatekeeper training known as GAT, produced significant and positive shifts in beliefs about suicide and mental health from before, to immediately after training. This was observed for all beliefs except Talking about suicide can cause suicide, for which there was relatively strong resistance to change. In terms of occupational differences, Professionals reported beliefs that showed greatest alignment with those of GAT. The beliefs of Managers and, to a slightly lesser extent, Clerical and Administrative workers, were also highly concordant with those of the GAT. It was these three occupational groups who showed greater shifts in beliefs, while Machinery Operators and Drivers, followed by Labourers and Technicians and Tradesworkers still showed favourable changes in beliefs—though of lesser magnitude.

The higher endorsement of GAT-aligned beliefs expressed by Professionals, Managers, and Clerical Workers, and greater amenability to change in these occupational groups is potentially associated with the higher levels of education, and or socioeconomic status in these occupational groups. This occupational patterning however, may also indicate a need for different messaging or a tailored intervention to those employed as Labourers, Machinery Operators and Drivers, and Technicians and Tradesworkers. The reduced tractability of beliefs and attitudes among this group is of particular interest, given that these occupations are known to be more vulnerable to suicidal behaviours [5].

The results suggest that the GAT intervention can successfully shift some beliefs regarding suicide and mental health at least in the short term. While at an individual level, these shifts may have little impact on an individual’s mental health or suicide risk, their importance at a population level should not be dismissed. Rose [26] notes that the population mean of a risk factor (such as suicide beliefs) is a predictor of the population distribution of that risk factor. Small shifts in individual risks or scores may mean little at an individual level, but may reduce the proportion of individuals at the high-risk tail end of the population. 

The results may also highlight the intransigence of some beliefs. *Talking about suicide can cause suicide* is emblematic of entrenched beliefs and attitudes regarding suicide contagion [1], as well as the highly stigmatising beliefs and attitudes that inhibit help-seeking by those contemplating suicide. Public or perceived stigma toward people who die by suicide is known to inhibit help-seeking and impede opportunities for assistance [27]. This finding therefore suggests that shifting this view should be a critical focus for suicide prevention programs, particularly among some occupational groups for whom there was lower endorsement of this belief.

This is also an important finding if we are attempting to promote discussion regarding the fear of causing suicide as an inhibiting factor in help-seeking behavior, and suggests a critical focus for programs to shift this view. The lack of change may suggest a need to: (a) modify the program to promote greater change; and or (b) modify the question to better capture change. Identifying which areas of belief, knowledge or attitudes need to be shifted by GAT (based on what is likely to engender behavioural change) should be given further thought, and this process should drive what measures/items are to be used in ongoing evaluation.

It is interesting to consider these shifts in the context of the questions about experiences and propensity to seek and provide assistance to others “doing it tough” (having a hard time). These questions were asked post-GAT and show that while the majority of respondents had previously helped someone, most respondents had not previously sought help. This higher likelihood of offering help (compared to seeking help) was also observed in responses to questions which asked about propensity to offer and seek future help when experiencing difficulties: while respondents rated themselves as extremely likely to offer help, they showed somewhat muted propensity to seek help. This may be reflective of perceived stigma toward mental health problems, as well as a perceived need to be self-reliant [28].

There are several limitations of these analyses. Firstly, while the delivery of GAT was standardised, we have no measure of how consistently the program was delivered, which could influence training outcomes. We attempted to control for this by adjusting for clustering by training session, however it is possible that this did not adequately adjust for variation in trainers/those administering GAT. We also note that in our occupational sub-analysis, there was a high level of missing data on occupation, which was largely due to occupation being coded as ‘other’. Missing occupation data was not randomly distributed across the sample: it was more prevalent in New South Wales and Queensland (40.0% and 39.4% respectively), and least common in Western Australia (22.4%). There was also slightly less missing occupation data among the youngest age group (32.9% compared to 36.2–37.9% for the other age groups), and more missing among women (53.2%). It is therefore possible that this missing data biased estimates in the occupational analysis. Related to this, as there was a high amount of missing occupational data, we did not adjust for occupation in the main analysis presented here, and it is possible that this introduced bias. The distribution of occupational groups varied by state (in Western Australia, there was a higher proportion of Technicians and Trades Workers, and in South Australia there was a higher proportion of Labourers) and age category (there was a higher proportion of Managers and Machinery Operators and Drivers in the older age group, and a higher proportion of Labourers in the younger age group) and gender (as is typical of the industry, women were over-represented in Managerial and Clerical/Administrative positions, but under-represented in most other positions). We were also unable to adjust for prior mental health, the omission of which may have biased the results. The fact that help-seeking intentions were not collected both pre- and post- GAT intervention means that we were unable to assess change in these. Further, these items do not enable us to distinguish between those who have not experienced a time when they or a friend were “doing it tough”, and those who have experienced such occasions but chose not to seek or offer help. It should also be noted that an increase in knowledge or change in attitudes amongst workers does not guarantee better mental health outcomes. While there are sound theoretical reasons to assume that removing barriers to help-seeking will allow earlier treatment, better support and improved outcomes, some have questioned whether psychoeducation alone is beneficial, and have highlighted the fact that increased mental health awareness is not without risks [29]. As such, it is important that future studies examine ongoing mental health and help seeking benefits related to the type of changes identified in this study. We also note that the lack of a control or comparison group limits assessment of the intervention success, and further to this, we had no longer term follow-up data to assess the enduring effect of the intervention. Future studies will seek to ascertain the longer-term effect of the intervention on attitudes and beliefs.

## 5. Conclusions

The results presented here indicate that the GAT program is associated with significant shifts in three out of four important beliefs about suicide and mental health that were examined. Beliefs that discussing suicide can cause suicide were less likely to be shifted, highlighting the challenge in shifting stigmatising beliefs and attitudes. Sub-analysis among different occupational groups indicated that those identified in other research as being at higher risk of suicide (Labourers, Machinery Operators and Drivers) [5] report beliefs that show poorer understanding of suicide, and are less amenable to belief change. This highlights the importance of workplace intervention programs such as MATES in Construction, and also emphasises the need for further work in understanding how to tailor interventions to these vulnerable groups.

## Figures and Tables

**Table 1:** Sample characteristics (total *n* = 20,125).

Characteristics	*n*	%
Age at training		
15–24 years	2972	14.8
25–34 years	6441	32.0
35–44 years	4816	23.9
45+ years	5896	29.3
Gender		
Female	1583	7.9
Male	18,542	92.1
State of training		
New South Wales	5456	27.1
Queensland	9214	45.8
Western Australia	1974	9.8
South Australia	3481	17.3
Year of training		
2016	4911	24.4
2017	13,694	68.0
2018	1520	7.6
Occupational Group *		
Managers	2146	16.7
Professionals	234	1.8
Technicians and Trade Workers	5171	40.2
Clerical and Administrative Workers	342	2.7
Machinery Operators and Drivers	1781	13.9
Labourers	3179	24.7

* *n* = 12,853, due to missing occupation.

**Table 2:** Attitudes and Experiences.

**Experiences of Suicide and Helping Behaviours**	**Yes**	
	***n***	**%**
Have you ever known someone who has died by or attempted suicide?	15,107	75.1
Have you ever sought help when you have been doing it tough?	7866	39.1
Have you ever helped someone else who is doing it tough?	15,628	77.7
**Help-Seeking and Help-Offering Propensity**	**Yes**	
	**Mean**	**SD**
If you were doing it tough in the future, how likely are you to ask for help?	3.74	0.92
If your mate was doing it tough in the future, how likely are you to offer help?	4.60	0.57

**Table 3:** Predictive margins for pre- and post -test beliefs, and mean differences in change in beliefs * #.

	Talking About Suicide Can Cause Suicide		People Considering Suicide Often Send Out Warning Signs or Invitations		Poor Mental Health is a Workplace Health and Safety Issue		The Construction Industry Must Do Something to Reduce Suicide Rates	
Predictive margin	3.62	3.62	2.67	2.25	1.82	1.67	1.72	1.57
Adjusted Mean difference (ß coefficient)	0.005		−0.429		−0.155		−0.151	
95% CI	−0.01, 0.02		−0.45, −0.41		−0.17, −0.14		−0.16, −0.14	
*p*-value	0.473		<0.001		<0.001		<0.001	

* On a scale of 1–5, where lower scores indicate greater agreement, and higher scores indicate greater disagreement. # Models adjusted for age category, gender, state of training, year of training, and also account for clustering by training session.

**Table 4:** Predictive margins for pre- and post-test beliefs, and mean differences in change in beliefs * for occupational groups #.

Occupational Group		Talking About Suicide Can Cause Suicide		People Considering Suicide Often Send Out Warning Signs or Invitations		Poor Mental Health is a Workplace Health and Safety Issue		The Construction Industry Must Do Something to Reduce Suicide Rates	
**Managers**		**Pre**	**Post**	**Pre**	**Post**	**Pre**	**Post**	**Pre**	**Post**
	**Predictive margin**	3.77	3.77	2.54	2.12	1.67	1.51	1.57	1.42
	**Adj. Mean difference (ß coefficient)**	0.06		−0.54		−0.19		−0.18	
	**95% CI**	0.02, 0.10		−0.59, −0.50		−0.23, −0.16		−0.21, −0.15	
	***p*-value**	0.004		<0.001		<0.001		<0.001	
**Professionals**		**Pre**	**Post**	**Pre**	**Post**	**Pre**	**Post**	**Pre**	**Post**
	**Predictive margin**	3.82	3.83	2.42	2.00	1.63	1.48	1.54	1.40
	**Adj. Mean difference (ß coefficient)**	0.01		−0.53		−0.18		−0.22	
	**95%CI**	−0.11, 0.14		−0.67, −0.39		−0.27, −0.09		−0.32, −0.13	
	***p*-value**	0.831		<0.001		<0.001		<0.001	
**Technicians and Trades Workers**		**Pre**	**Post**	**Pre**	**Post**	**Pre**	**Post**	**Pre**	**Post**
	**Predictive margin**	3.63	3.63	2.70	2.28	1.85	1.69	1.74	1.59
	**Adj. Mean difference (ß coefficient)**	−0.01		−0.43		−0.17		−0.14	
	**95% CI**	−0.03, 0.01		−0.45, −0.40		−0.19, −0.15		−0.16, −0.13	
	***p*-value**	0.390		<0.001		<0.001		<0.001	
**Clerical and Administrative Workers**		**Pre**	**Post**	**Pre**	**Post**	**Pre**	**Post**	**Pre**	**Post**
	**Predictive margin**	3.69	3.69	2.55	2.13	1.74	1.59	1.60	1.46
	**Adj. Mean difference (ß coefficient)**	0.10		−0.55		−0.20		−0.16	
	**95% CI**	−0.00, 0.19		−0.67, −0.43		−0.30, −0.10		−0.25, −0.07	
	***p*-value**	0.048		<0.001		<0.001		=0.001	
**Machinery Operators and Drivers**		**Pre**	**Post**	**Pre**	**Post**	**Pre**	**Post**	**Pre**	**Post**
	**Predictive margin**	3.52	3.52	2.75	2.33	1.91	1.76	1.83	1.68
	**Adj. Mean difference (ß coefficient)**	−0.01		−0.42		−0.14		−0.16	
	**95% CI**	−0.05, 0.03		−0.46, −0.37		−0.18, −0.11		−0.19, −0.12	
	***p*-value**	0.722		<0.001		<0.001		<0.001	
**Labourers**		**Pre**	**Post**	**Pre**	**Post**	**Pre**	**Post**	**Pre**	**Post**
	**Predictive margin**	3.53	3.53	2.70	2.28	1.87	1.71	1.77	1.63
	**Adj. Mean difference (ß coefficient)**	−0.03		-0.37		−0.12		−0.13	
	**95% CI**	−0.06, 0.00		−0.40, −0.34		−0.15, −0.10		−0.15, −0.11	
	***p*-value**	0.067		<0.001		<0.001		<0.001	

* On a scale of 1–5, where lower scores indicate greater agreement, and higher scores indicate greater disagreement. # Models adjusted for age category, gender, state of training, year of training, and also account for clustering by training session. Given high missing data on occupation, these analyses were conducted on a smaller sample of those with complete occupation data (*n* = 12,853).

## Supplementary Materials

The questionnaire is available online at http://www.mdpi.com/1660-4601/15/10/2106/s1.

## References

[1] World Health Organization (2014). Preventing Suicide: A Global Imperative.

[2] Windfuhr K., Kapur N., O’Connor R., Pratt S., Gordon J. (2011). International Perspectives on the Epidemiology and Aetiology of Suicide and Self-Harm. International Handbook of Suicide Prevention: Research, Policy and Practice.

[3] Milner A., Spittal M.J., Pirkis J., LaMontagne A.D. (2013). Suicide by occupation: Systematic review and meta-analysis. Br. J. Psychiatr..

[4] Milner A., LaMontagne A.D., Burke R.J., Cooper C. (2018). Suicide prevalence and suicide prevention in the workplace. Violence and Abuse in and around Organisations.

[5] Milner A., Niven H., LaMontagne A. (2014). Suicide by occupational skill level in the Australian construction industry: Data from 2001 to 2010. Aust. N. Z. J. Public Health.

[6] Rutz W. (2004). Suicidal behaviour: Comments, advancements, challenges. A European perspective. World Psychiatry.

[7] Milner A., McClure R., De Leo D. (2012). Socio-economic determinants of suicide: An ecological analysis of 35 countries. Soc. Psychiatry Psychiatr. Epidemiol..

[8] Pirkis J., Mok K., Robinson J., Nordentoft M., O’Connor R., Pirkis J. (2016). Media influences on suicidal thoughts and behaviors. International Handbook of Suicide Prevention.

[9] Milner A., Maheen H., Currier D., LaMontagne A.D. (2017). Male suicide among construction workers in Australia: A qualitative analysis of the major stressors precipitating death. BMC Public Health.

[10] Czyz E.K., Horwitz A.G., Eisenberg D., Kramer A., King C.A. (2013). Self-reported barriers to professional help seeking among college students at elevated risk for suicide. J. Am. Coll. Health.

[11] Alexander L., Link B. (2003). The impact of contact on stigmatizing attitudes toward people with mental illness. J. Ment. Health.

[12] Milner A., Law P.C.F., Mann C., Cooper T., Witt K., LaMontagne A.D. (2018). A smart-phone intervention to address mental health stigma in the construction industry: A two-arm randomised controlled trial. Soc. Sci. Med. Popul. Health.

[13] Han J., Batterham P.J., Calear A.L., Wu Y., Xue J., van Spijker B.A. (2018). Development and pilot evaluation of an online psychoeducational program for suicide prevention among university students: A randomised controlled trial. Internet Interv..

[14] Hom M.A., Stanley I.H., Joiner T.E. (2015). Evaluating factors and interventions that influence help-seeking and mental health service utilization among suicidal individuals: A review of the literature. Clin. Psychol. Rev..

[15] Zalsman G., Hawton K., Wasserman D., van Heeringen K., Arensman E., Sarchiapone M., Carli V., Höschl C., Barzilay R., Balazs J. (2016). Suicide prevention strategies revisited: 10-year systematic review. Lancet Psychiatry.

[16] Isaac M., Elias B., Katz L.Y., Belik S.L., Deane F.P., Enns M.W., Sareen J. (2009). Gatekeeper training as a preventative intervention for suicide: A systematic review. Can. J. Psychiatry.

[17] Cross W., Matthieu M.M., Cerel J., Knox K.L. (2007). Proximate outcomes of gatekeeper training for suicide prevention in the workplace. Suicide Life-Threat. Behav..

[18] Australian Institute for Suicide Research and Prevention (2006). Suicide in Queensland’s Commercial Building and Construction Industry: An Investigation of Factors Associated with Suicide and Recommendations for the Prevention of Suicide.

[19] Gullestrup J., Lequertier B., Martin G. (2011). MATES in construction: Impact of a multimodal, community-based program for suicide prevention in the construction industry. Int. J. Environ. Res. Public Health.

[20] Martin G., Swannell S., Milner A., Gullestrup J. (2016). Mates in construction suicide prevention program: A five year review. J. Commun. Med. Health Educ..

[21] Australian Bureau of Statistics (2013). ANZSCO—Australian and New Zealand Standard Classification of Occupations.

[22] StataCorp L.P. (2017). Stata Statistical Software: Release 15.

[23] Liang K.Y., Zeger S.L. (1986). Longitudinal data analysis using generalized linear models. Biometrika.

[24] Fitzmaurice G.M., Laird N.M., Ware J.H. (2011). Applied Longitudinal Analysis.

[25] Rabe-Hesketh S., Skrondal A. (2008). Multilevel and Longitudinal Modeling Using Stata.

[26] Rose G. (1985). Sick individuals and sick populations. Int J Epidemiol.

[27] Calear A.L., Batterham P.J., Christensen H. (2014). Predictors of help-seeking for suicidal ideation in the community: Risks and opportunities for public suicide prevention campaigns. Psychiatry Res..

[28] Latalova K., Kamaradova D., Prasko J. (2014). Perspectives on perceived stigma and self-stigma in adult male patients with depression. Neuropsychiatr. Dis. Treat..

[29] Wessely S., Bryant R.A., Greenberg N., Earnshaw M., Sharpley J., Hughes J.H. (2008). Does psychoeducation help prevent post traumatic psychological distress?. Psychiatry Interpers. Boil. Process..

