# Supplementary material for: Shifting Beliefs about Suicide: Pre-Post Evaluation of the Effectiveness of a Program for Workers in the Construction Industry
Source: Int J Environ Res Public Health. 2018 Sep 25;15(10):2106. doi: 10.3390/ijerph15102106 (PMC6211080; doi:10.3390/ijerph15102106)
Supplement: Supplementary file 1 [file ijerph-15-02106-s001.pdf]

FEEDBACK FORM

Please provide your details and respond to these statements **BEFORE** completing General Awareness Training (GAT).

YOUR DETAILS

Gender: ◯ Male ◯ Female D.O.B. ____________

Name ____________

Email ____________

Employer ____________

Occupation ____________

Phone ____________

Mobile ____________

Address ____________

____

____________ Postcode ____________

Site ____________

Have you had previous training by MIC?

◯ GAT ◯ Connector ◯ A.S.I.S.T.

MIC may send you important information about your health and wellbeing by email or post

◯ Please tick here if you do not want to receive any MIC information or material

Please note: under no circumstances will MIC provide personal information to third persons without your express consent.

To you want this to read.... by email or post or text.....?

Please circle the number to indicate your level of agreement with each of the statements below:

1 = Strongly Disagree 2 = Disagreee 3 = Neither agree nor disagreee 4 = Agree 5 = Strongly Agree

	1	2	3	4	5
I am familiar with Mates in Construction and the work that they do	❒	❒	❒	❒	❒
Talking openly about suicide can prevent suicide	❒	❒	❒	❒	❒
If my workmate was going through a difficult time feeling upset or thinking about suicide, I think I would notice	❒	❒	❒	❒	❒
If my mate was going through a difficult time feeling upset or was thinking about suicide, I would be willing to offer help	❒	❒	❒	❒	❒
If my workmate was going through a difficult time feeling upset or thinking about suicide I would know how to connect him/her to appropriate help	❒	❒	❒	❒	❒
My current worksite supports good mental health and well-being	❒	❒	❒	❒	❒
If I was going through a difficult time, feeling upset, or was thinking about suicide, I would be willing to seek help	❒	❒	❒	❒	❒

I WOULD SEEK HELP FROM:

Please circle the number to indicate how likely you would be to seek help from the options listed.

1 = Extremely Unlikely 2 = Unlikely 3 = Neutral 4 = Likely 5 = Extremely Likely

	1	2	3	4	5
Intimate partner	❒	❒	❒	❒	❒
Close family	❒	❒	❒	❒	❒
Friend	❒	❒	❒	❒	❒
Workmate	❒	❒	❒	❒	❒
A supervisor	❒	❒	❒	❒	❒
My doctor	❒	❒	❒	❒	❒
Mental health professional (Psychologist, social worker, counsellor)	❒	❒	❒	❒	❒
A telephone helpline (e.g. Lifeline)	❒	❒	❒	❒	❒
MIC worker/Connector	❒	❒	❒	❒	❒
Minister or religious leader	❒	❒	❒	❒	❒
I would not seek help from anyone	❒	❒	❒	❒	❒
I would seek help from another (please list)	❒	❒	❒	❒	❒

So far today, the best way to describe how I'm feeling emotionally/mentally is ...

Very poor ❒ poor ❒ ok ❒ good ❒ very good ❒

INVITATIONS

Things you might know

- Family crisis
- Separation, divorce or relationship break down
- Recent loss
- Anniversary of the death of a friend or relative
- Drug and/or alcohol dependence
- Mental illness
- Financial crisis

OFTEN ABOUT **LOSS**

Things you might notice

- Moody
- Distant
- Attitude change
- Increased use of drugs and/or alcohol
- Carelessness and/or risk taking
- Giving away possessions
- Putting affairs in order
- Preparing to go away

NOTICE **CHANGES**

Things you might hear

- Lonely
- Hopeless
- Escape
- Depressed
- Guilt
- Giving up

OFTEN **SUBTLE**

PLEASE DETACH AND FOLD YOUR POCKET GUIDE

IF YOU THINK SOMEONE COULD BE THINKING ABOUT SUICIDE, YOU SHOULD ALWAYS ACT AS IF THEY ARE.
TALK TO A CONNECTOR OR CALL 1300 642 111

Please provide your details and respond to these statements **AFTER** completing General Awareness Training (GAT).

Please circle the number to indicate your level of agreement with each of the statements below:

1 = Strongly Disagree 2 = Disagreee 3 = Neither agree nor disagreee 4 = Agree 5 = Strongly Agree

	1	2	3	4	5
Talking openly about suicide can prevent suicide	❐	❐	❐	❐	❐
If my workmate was going through a difficult time feeling upset or thinking about suicide, I think I would notice	❐	❐	❐	❐	❐
If my mate was going through a difficult time feeling upset or was thinking about suicide, I would be willing to offer help	❐	❐	❐	❐	❐
If my workmate was going through a difficult time feeling upset or thinking about suicide I would know how to connect him/her to appropriate help	❐	❐	❐	❐	❐
My current worksite supports good mental health and well-being	❐	❐	❐	❐	❐
If I was going through a difficult time, feeling upset, or was thinking about suicide, I would be willing to seek help	❐	❐	❐	❐	❐

I WOULD SEEK HELP FROM:

Please circle the number to indicate how likely you would be to seek help from the options listed.

1 = Extremely Unlikely 2 = Unlikely 3 = Neutral 4 = Likely 5 = Extremely Likely

	1	2	3	4	5
Intimate partner	❐	❐	❐	❐	❐
Close family	❐	❐	❐	❐	❐
Friend	❐	❐	❐	❐	❐
Workmate	❐	❐	❐	❐	❐
A supervisor	❐	❐	❐	❐	❐
My doctor	❐	❐	❐	❐	❐
Mental health professional (Psychologist, social worker, counsellor)	❐	❐	❐	❐	❐
A telephone helpline (e.g. Lifeline)	❐	❐	❐	❐	❐
MIC worker/Connector	❐	❐	❐	❐	❐
Minister or religious leader	❐	❐	❐	❐	❐
I would not seek help from anyone	❐	❐	❐	❐	❐
I would seek help from another not listed above (list in the space provided)	❐	❐	❐	❐	❐

So far today, the best way to describe how I'm feeling emotionally/mentally is …

Very poor ❐ poor ❐ ok ❐ good ❐ very good ❐

Would you like a follow up call from a MIC Field Officer?

◯ Yes ◯ No

Are you interested in being involved in the project as a Connector?

◯ Yes ◯ No

SUPPORTED BY:

SUPPORTING THE INDUSTRY

MATES IN CONSTRUCTION (AUST) Ltd - ABN 99 166 347 539

PLEASE DETACH AND FOLD YOUR POCKET GUIDE

General Awareness Trained – keep an eye on your mates

▼

If you're not sure how to help your mate then talk to a Connector

▼

A Connector is a mate who can keep you safe while connecting you to help

▼

If the issue relates to suicide

▼

A suicide first-aid trained person

IMPORTANT NUMBERS

MIC HELPLINE
1300 642 111

EMERGENCY SERVICES 000
POLICE 131 444
LIFELINE 13 11 14

SUICIDE CALLBACK SERVICE
1300 659 467

MENS LINE AUSTRALIA
1300 78 99 78

SPONSORED BY BLUEY
Innovative products, service and value
bluey.com.au

MATES in Construction

POCKET GUIDE
